# Pattern and Features of Pediatric Endocrinology Referrals: A Retrospective Study in a Single Tertiary Center in Italy

**DOI:** 10.3389/fped.2020.580588

**Published:** 2020-10-02

**Authors:** Eleonora Bellotto, Lorenzo Monasta, Maria Chiara Pellegrin, Benedetta Bossini, Gianluca Tamaro, Maria Sole Conte, Elena Faleschini, Egidio Barbi, Gianluca Tornese

**Affiliations:** ^1^University of Trieste, Trieste, Italy; ^2^Institute for Maternal and Child Health IRCCS “Burlo Garofolo”, Trieste, Italy

**Keywords:** endocrinologic diseases, referral, visits and budgets of health, epidemioiogy, health service access, growth, thyroid, puberty

## Abstract

**Introduction:** The knowledge of the pattern and the features of pediatric endocrinology referrals is crucial to optimize resources and guide public health interventions. We explored the numbers and the reasons for referral to a pediatric endocrinology outpatient clinic and investigated their features in terms of assignment of priority ranks, sex, age differences, the prevalence of pathological findings among referred cases, and the agreement among referrals, final diagnosis, treatment, and follow-up.

**Methods:** Retrospective study with data collection for pediatric endocrinology first visits between November 2012 and February 2019 in a tertiary center.

**Results:** A total of 1930 first visits were performed with an overall number of referrals of 2,165, and an increasing trend over the years. The most frequent referral reasons were slow growth, precocious puberty, and obesity; 14% of visits were classified as “urgent” (<7 days), 35% as “deferrable” (<30 days), and 51% as “planned” (<180 days). Sex and age differences among referrals were detected, with criticality in the appropriate timing for referral. Thirty-eight percent of patients had pathological findings. In 4% of the cases the final diagnosis was not concordant with the reason for referral. Treatment was prescribed in 35% of cases, and 67% returned at least for one follow-up visit.

**Conclusion:** The study highlighted the need to target medical education of primary care on the definition of priority ranks, the need for more extended observation periods for subclinical or para-physiological conditions, the appropriate timing for referral, based on the definition of conditions or the best window of intervention.

## Introduction

Pediatric endocrinology deals with the diagnosis and treatment of children with diseases of the endocrine system, which may affect growth, development, and reproduction (1). Concerns about endocrinological problems are a common cause of parents' anxiety (2, 3). Therefore, referrals from primary care physicians to pediatric endocrinologists represent a first critical step in the specialist care coordination (4–6): while over-referrals lead to inefficient use of resources and poor patient experience, high-quality referrals increase the likelihood that the specialist can provide timely and efficient care (7).

In Italy, primary care is free of charge and is provided by family pediatricians (FP) up to 14–16 years (8). These physicians have a “gatekeeping” role, being responsible for referring patients to specialist consultations if needed. In the referral, apart from the reason for the visit, a priority rank should be assigned by the FP (“urgent,” “deferrable” or “planned,” meaning that the visit must be performed within 7, 30, or 180 days, respectively) based on his/her judgment, with no fixed rules.

The knowledge of the pattern and the features of pediatric endocrinology referrals is crucial to guide public health interventions, both to organize outpatient clinics and to address continuing medical education of primary care and, consequently, avoid inappropriate referrals. To date, no study has extensively evaluated this issue, taking into account the entire field of pediatric endocrinology.

This study aimed to explore the numbers and the reasons for referral to a pediatric endocrinology outpatient clinic in a tertiary center, and to investigate their features in terms of assignment of priority ranks, sex and age differences, the prevalence of pathological findings among referred cases, and the agreement between referrals, final diagnosis treatment, and follow-up.

## Materials and Methods

We conducted a retrospective study at the Institute for Maternal and Child Health IRCCS “Burlo Garofolo” in Trieste, Italy, a tertiary hospital and research institute that serves as a pediatric referral center for the province of Trieste, and as national reference hospital.

All records of children and adolescents addressed to the pediatric endocrinology outpatient clinic for a new endocrine condition were reviewed. A maximum of 15 visits per week (first visits/new referrals or follow-up visits, according to needs and bookings) were performed in the outpatient department from three consultants (MCP, EF, GT); overall, a maximum of 780 visits per year could be performed in the outpatient clinics.

Information was obtained from web-based platforms adopted by the Regional Health System. The “*Cup Web”* platform (online booking system for specialist services) was used to collect data about all first visits performed between November 2012 (when the platform was first introduced) and February 2019. The “*G2 clinico”* platform (management system specialist activities) was employed to access all patients' diagnostic and therapeutic data. Information retrieved included age at presentation, gender, priority rank, reason(s) for referral, whether endocrinologist prescribed diagnostic investigations, therapies, and follow-ups (according to internal protocols), whether a final diagnosis was given and whether it was concordant with the primary reason for referral. Referral reasons were considered as items if they resulted in at least an absolute frequency of five visits. Twelve reasons were defines as follows: “Adrenal,” “Bone,” “Dyslipidemias,” “Glucose metabolism,” “Growth,” “Gynecology,” “Pituitary gland,” “Puberty,” “Syndromes,” “Thyroid,” “Weight,” and “Others” (see Table 1).

**Table 1:** Distribution of referral reasons, divided in 12 cumulative groups in order of frequency.

**Referral reasons**	**Total**				**Females**			**Males**		
	***n***	**% of**	**% of**	**Median age (IQR)**	***n***	**% of**	**Median age (IQR)**	***n***	**% of**	**Median age (IQR)**
		**group**	**total**			**group**			**group**	
**Growth**	566		26.1	11.3 (8.2–13.1)	234	41.3%	11.0 (7.7–12.4)	332	58.7%	11.7 (8.4–13.5)
Growth delay	401	70.8	18.5	11.2 (7.9–13.0)	163	28.8%	10.9 (6.4–12.4)	238	42.0%	11.5 (8.3–13.5)
Short stature	114	20.1	5.3	11.2 (8.7–13.2)	50	8.8%	11.3 (9.8–13.1)	64	11.3%	11.1 (7.7–13.3)
Growth check-up	29	5.1	1.3	12.3 (11.1–14.5)	8	1.4%	11.5 (10.3–12.5)	21	3.7%	12.7 (11.3–15.6)
Tall stature	13	2.3	0.6	8.3 (6.8–13.2)	7	1.2%	7.8 (2.7–8.4)	6	1.1%	12.0 (8.3–14.8)
Stunted growth	9	1.6	0.4	12.9 (10.0–14.2)	6	1.1%	10.8 (2.3–14.2)	3	0.5%	13.2 (12.9–17.1)
**Puberty**	456		21.1	8.5 (7.1–10.6)	297	65.1%	7.9 (6.2–8.9)	159	34.9%	11.9 (9.0–13.8)
Precocious puberty	231	50.7	10.7	8.2 (7.6–9.2)	187	41.0%	8.2 (7.5–9.0)	44	9.6%	9.4 (8.5–10.2)
Precocious thelarche	83	18.2	3.8	3.08 (1.4–7.3)	83	18.2%	3.1 (1.4–7.3)			
Gynecomastia	52	11.4	2.4	13.7 (12.6–14.7)				52	11.4%	13.7 (12.6–14.7)
Pubertal delay	29	6.4	1.3	14.02 (13.3–14.6)	9	2.0%	13.6 (13.3–15.1)	20	4.4%	14.1 (13.1–14.5)
Micropenis	18	3.9	0.8	10.6 (8.2–11.9)				18	3.9%	10.6 (9.2–11.9)
Precocious menarche	13	2.9	0.6	9.6 (9.2–10.7)	13	2.9%	9.7 (9.2–10.7)			
Cryptorchidism	9	2.0	0.4	4.6 (2.2–6.8)				9	2.0%	4.6 (2.2–6.8)
Hypogonadism	9	2.0	0.4	10.6 (7.0–11.5)				9	2.0%	10.6 (7.0–11.6)
Others	12	2.6	0.6	9.0 (6.6–12.5)	5	1.1%	7.1 (6.1–8.7)	7	1.5%	12.4 (7.9–12.7)
**Thyroid**	443		20.5	12.2 (8.5–15.0)	295	66.6%	12.4 (8.8–15.0)	148	33.4%	12.1 (6.4–14.6)
Thyroiditis	126	28.4	5.8	13.2 (10.1–15.0)	93	21.0%	13.2 (10.1–14.9)	33	7.4%	12.8 (10.0–15.5)
Hypothyroidism	104	23.5	4.8	12.6 (8.6–15.8)	62	14.0%	13.7 (9.1–16.5)	42	9.5%	11.2 (5.9–14.0)
Subclinical hypothyroidism	103	23.3	4.8	11.3 (8.7–14.4)	64	14.4%	10.6 (8.6–13.9)	39	8.8%	12.8 (8.8–14.7)
Hyperthyroidism	22	5.0	1.0	12.8 (10.6–14.8)	15	3.4%	11.8 (10.5–14.1)	7	1.6%	14.2 (12.7–15.6)
Congenital hypothyroidism	21	4.7	1.0	1.5 (0.4–5.2)	10	2.3%	2.5 (0.4–6.9)	11	2.5%	1.3 (0.4–3.4)
Nodule	18	4.1	0.8	12.9 (5.9–16.0)	13	2.9%	12.5 (6.8–15.3)	5	1.1%	15.1 (5.1–16.1)
fT3 increased	14	3.2	0.6	10.7 (5.9–13.9)	11	2.5%	8.4 (4.4–16.4)	3	0.7%	13.1 (11.6–13.9)
Dysthyroidism	12	2.7	0.6	9.3 (4.1–13.9)	10	2.3%	7.3 (3.7–13.4)	2	0.5%	13.6 (12.9–14.3)
Familiarity	12	2.7	0.6	14.0 (12.1–15.5)	9	2.0%	14.8 (13.0–15.0)	3	0.7%	12.2 (12.0–19.9)
Goiter	7	1.6	0.3	13.4 (11.4–15.4)	5	1.1%	13.9 (13.4–15.4)	2	0.5%	12.3 (11.4–13.1)
Cyst	4	0.9	0.2	7.6 (6.5–10.8)	3	0.7%	8.8 (6.7–12.7)	1	0.2%	6.3
**Weight**	315		14.5	11.0 (8.6–13.6)	154	48.9%	10.6 (7.9–13.7)	161	51.1%	11.5 (9.6–13.3)
Obesity	184	58.4	8.5	11.2 (8.5–13.7)	92	29.2%	10.2 (7.6–14.0)	92	29.2%	11.7 (9.6–13.2)
Overweight	119	37.8	5.5	10.9 (9.2–13.3)	59	18.7%	10.6 (8.0–12.5)	60	19.0%	11.3 (10.2–13.5)
Underweight	12	3.8	0.6	11.9 (1.5–16.2)	3	1.0%	16.2 (10.8–17.6)	9	2.9%	2.7 (1.1–15.5)
**Adrenal**	136		6.3	8.3 (6.8–10.3)	108	79.4%	8.1 (6.8–10.7)	28	20.6%	8.7 (6.6–9.7)
Precocious adrenarche	65	47.8	3.0	7.9 (6.8–8.7)	53	39.0%	7.5 (6.8–8.5)	12	8.8%	8.7 (8.2–9.1)
Hirsutism	19	14.0	0.9	13.8 (12.3–16.5)	19	14.0%	13.8 (12.3–16.5)			
Hypertrichosis	16	11.8	0.7	9.3 (5.2–13.8)	13	9.6%	8.5 (5.6–14.7)	3	2.2%	9.8 (3.2–12.1)
Precocious pubarche	16	11.8	0.7	7.9 (4.5–9.2)	12	8.8%	7.9 (5.6–9.7)	4	2.9%	4.6 (0.5–8.9)
Adult body odor	9	6.6	0.4	6.7 (6.4–6.8)	6	4.4%	6.7 (6.5–6.8)	3	2.2%	6.5 (0.5–8.5)
Hypercortisolism	6	4.4	0.3	14.0 (12.4–14.4)	2	1.5%	13.0 (12.4–13.6)	4	2.9%	14.4 (11.7–15.1)
Others	5	3.7	0.2	8.2 (7.9–11.0)	3	2.2%	8.2 (7.9–11.0)	2	1.5%	8.9 (0.7–17.1)
**Gynecology**	75		3.5	15.1 (13.7–16.2)	75	100.0%	15.1 (13.8–16.2)			
Menstrual irregularities	30	40.0	1.4	14.5 (124–16.6)	30	40.0%	14.5 (12.4–16.6)			
Secondary amenorrhea	17	22.7	0.8	15.0 (14.4–15.4)	17	22.7%	15.0 (14.5–15.5)			
Primary amenorrhea	16	21.3	0.7	15.6 (14.6–16.5)	16	21.3%	15.6 (14.6–16.5)			
PCOS	10	13.3	0.5	15.5 (14.9–15.9)	10	13.3%	15.5 (14.9–16.0)			
Others	2	2.7	0.1	13.0 (12.2–13.9)	2	2.7%	13.0 (12.2–13.9)			
**Dyslipidemias**	52		2.4	10.0 (7.5–124)	26	50.0%	10.3 (7.8–13.1)	26	50.0%	9.8 (6.3–12.1)
Hypercholesterolemia	44	84.6	2.0	9.5 (6.8–12.1)	23	44.2%	10.9 (7.6–14.2)	21	40.4%	8.9 (4.1–10.7)
Others	8	15.4	0.4	11.6 (9.3–13.7)	3	5.8%	8.5 (5.5–10.6)	5	9.6%	12.7 (12.6–14.7)
**Syndromes**	31		1.4	8.4 (1.9–11.3)	13	41.9%	2.3 (1.2–9.8)	18	58.1%	9.9 (7.7–11.6)
Klinefelter syndrome	13	41.9	0.6	8.4 (5.3–10.6)				13	41.9%	8.4 (5.4–10.6)
Others	18	58.1	0.8	8.6 (1.4–11.3)	13	41.9%	2.3 (1.2–9.8)	5	16.1%	11.3 (10.7–12.7)
**Glucose metabolism**	28		1.3	11.5 (7.2–13.9)	8	28.6%	9.4 (4.4–14.2)	20	71.4%	11.5 (9.2–13.4)
Hyperglyaemia	18	64.3	0.8	12.3 (8.3–15.0)	5	17.9%	13.5 (5.3–14–3)	13	46.4%	12.0 (10.1–15.0)
Hypoglycemia	10	35.7	0.5	10.7 (3.7–11.5)	3	10.7%	3.7 (3.4–14.2)	7	25.0%	11.0 (6.4–11.5)
**Pituitary gland**	16		0.7	14.7 (12.4–16.5)	12	75.0%	14.7 (13.0–16.5)	4	25.0%	14.3 (9.5–16.5)
Adenoma	6	37.5	0.3	14.7 (13.9–16.1)	5	31.3%	14.7 (13.9–14.7)	1	6.3%	16.1
Hyperprolactinemia	6	37.5	0.3	14.9 (12.5–16.9)	5	31.3%	16.0 (13.8–16.9)	1	6.3%	12.5
Others	4	25.0	0.2	11.0 (7.0–16.0)	2	12.5%	11.2 (7.4–15.0)	2	12.5%	11.7 (6.5–16.9)
**Bone**	7		0.3	2.9 (1.5–14.3)	7	100.0%	2.9 (1.4–14.3)			
**Others**	40		1.8	11.8 (9.9–14.4)	24	60.0%	13.2 (9.9–14.6)	16	40.0%	10.9 (9.9–13.5)
**Total**	2,165		100.0	10.5 (7.6–13.4)	1253	57.9%	9.7 (7.2–13.2)	912	42.1%	11.3 (8.5–13.6)

Ethical Committee approval was not requested since General Authorization to Process Personal Data for Scientific Research Purposes (Authorization no. 9/2014) declared that retrospective archive studies that use ID codes, preventing the data from being traced back directly to the data subject, do not need ethics approval (9). Informed consent was signed by parents at first visit, in which they agree that “clinical data may be used for clinical research purposes, epidemiology, study of pathologies and training, with the objective of improving knowledge, care and prevention.”

Statistical analyses were mainly descriptive. Data are presented as frequencies and percentages, or as median and interquartile ranges (IQRs). Mann-Whitney rank-sum tests and Two-tailed Fisher exact tests were executed to study the relations between variables. Analyses were performed with Stata/IC 14.2 (StataCorp LLC, College Station, USA).

## Results

A total of 1930 first visits were carried out over the study period for 1,893 patients, 1,086 females (median age 9.7 years, IQR 7.2–13.2), 807 males (median age 11.3 years, IQR 8.5–13.6). Thirty-seven patients had two or more visits for different reasons, while 235 patients had a single visit for multiple referrals (e.g., “overweight” and “subclinical hypothyroidism”). For this reason, the overall number of referrals was 2,165.

### Referral Reasons and Trends Over Years

**Figure 1:**
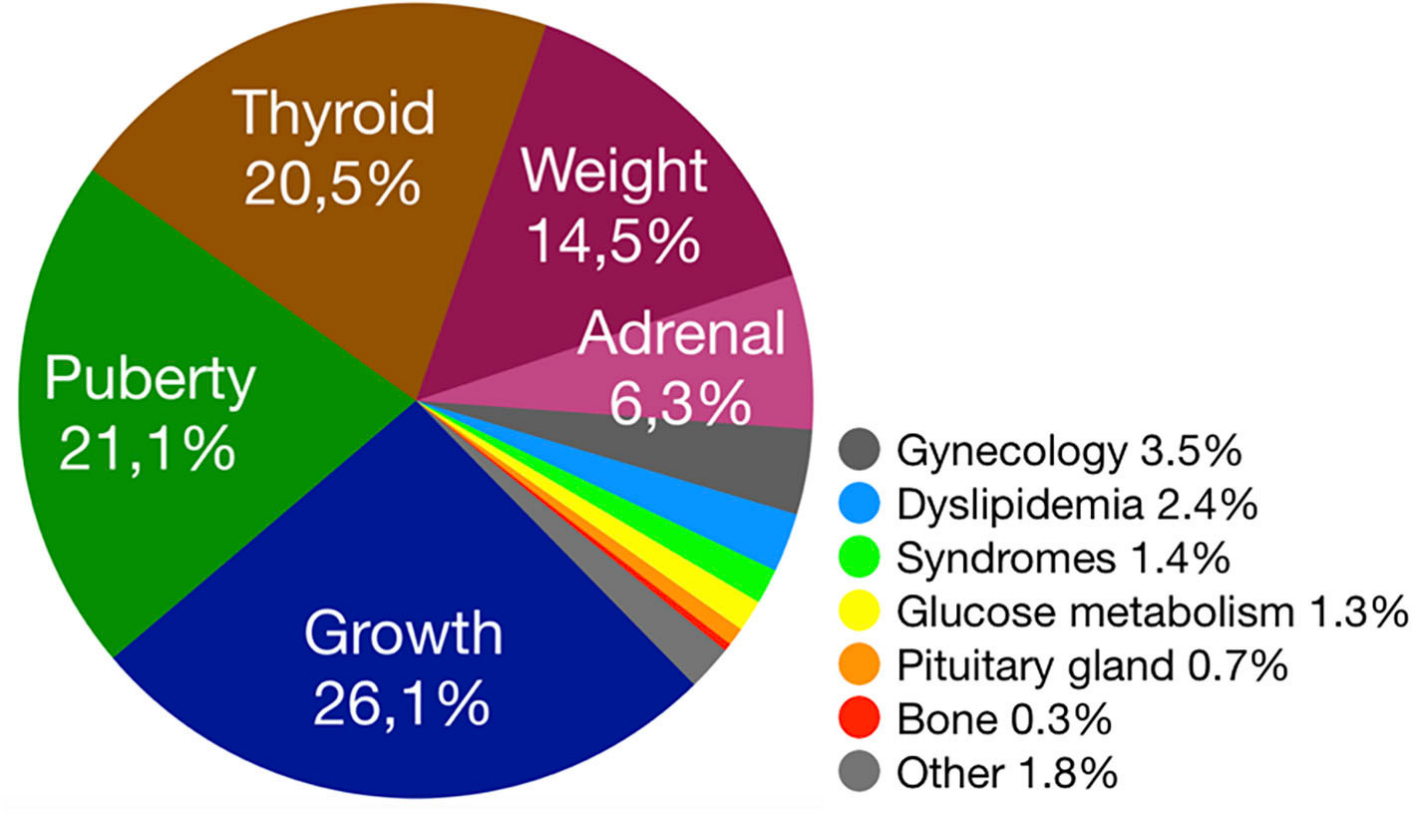
Distribution of referral reasons per main cumulative groups.

Considering cumulative groups, the most frequent reason for referrals were “Growth” (*n* = 566, 26.1%), “Puberty” (*n* = 456, 21.1%), “Thyroid” (*n* = 443, 20.5%), “Weight” (*n* = 315, 14.5%) and “Adrenal” (*n* = 136, 6.3%) (Table 1; Figure 1).

When considering single referral reasons, the most frequent were slow growth (*n* = 401, 18.5%), *p*recocious puberty (*n* = 231, 10.7%), obesity (*n* = 184, 8.5%) and thyroiditis (*n* = 126, 5.8%) (Table 1).

As regard the trend of new referrals over time, there was an overall upward trend, with 255 visits in 2013 (32% on overall outpatients clinics) and 466 visits in 2018 (58%). The most significant percentage increases were detected in “Weight” (+240%), “Gynecology” (+200%) and “Adrenal” (+191%) groups (excluding groups for which absolute frequency was low). The most significant numerical increases were found in the “Weight” (+72 visits), “Growth” (+40 visits) and “Puberty” (+30 visits) groups. A dramatic increase from 2017 to 2018 in terms of referrals for “Weight” and “Gynecology” was noted (Table 2).

**Table 2:** Numeric variations of referrals during the 2013–2018 period (only entire year, with data available from January to December, were included).

	**TOTAL**	**2013**	**2014**	**2015**	**2016**	**2017**	**2018**	**Increase in % from 2013 to 2018**	**Increase in *n* from 2013 to 2018**
Growth	537	78	59	89	96	97	118	51%	40
Puberty	436	45	71	81	85	79	75	67%	30
Thyroid	424	67	60	72	86	63	76	13%	9
Weight	302	30	38	38	32	62	102	240%	72
Adrenal	124	11	17	19	20	25	32	191%	21
Gynecology	72	8	9	10	12	9	24	200%	16
Dyslipidemias	49	1	5	10	9	11	13	1,200%	12
Syndromes	30	6	2	2	6	8	6	0%	0
Glucose metabolism	28	4	4	3	6	4	7	75%	3
Pituitary Gland	16	1	1	3	6	2	3	200%	2
**Total**	**2,018**	**251**	**266**	**327**	**358**	**360**	**456**	82%	205

### Features of Referrals

#### Assignment of Priority Ranks

The priority rank indicated in the referral was “urgent” in 14% of cases (*n* = 299), “deferrable” in 35% (*n* = 751) and “planned” in 51% (*n* = 1,115). Over the study period, there was a significant and progressive rise in “deferrable” visits (from 40 in 2013 to 263 in 2018), compared to other priority ranks (“urgent” from 36 to 39, “planned” from 179 to 164) (*p* < 0.01).

The age at referral was significantly higher in “planned” (11.45 years, IQR 8.28–14.11) compared to “deferrable” (9.89 years, IQR 7.44–12.73, *p* < 0.01) and to “urgent” visits (9.34 years, IQR 6.93–12.93, *p* < 0.01).

**Figure 2:**
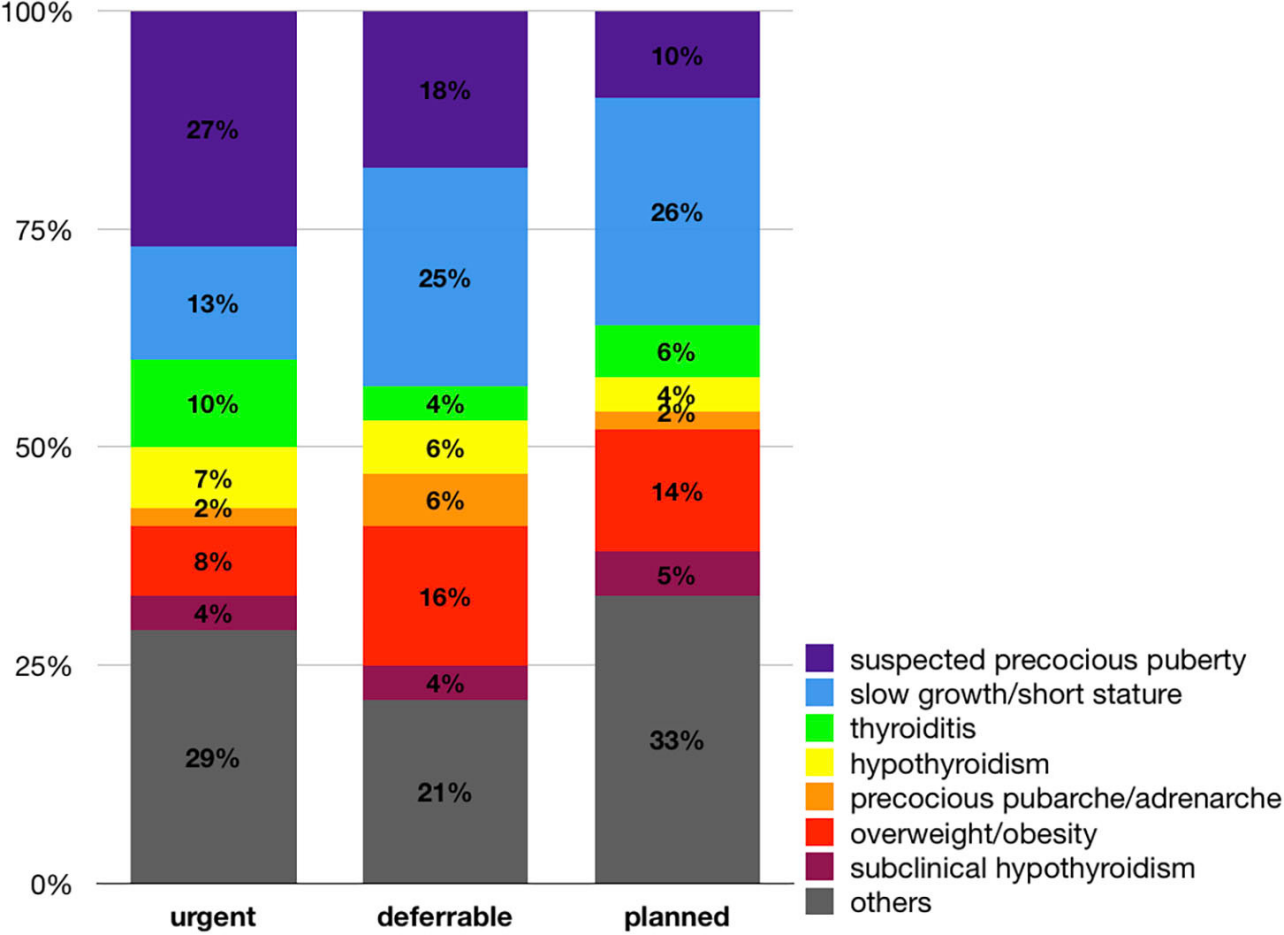
Distribution of referral reasons according to priority ranks. Visits for suspected precocious puberty were more frequent among “urgent” referrals compared to “deferrable” and “planned” ones (*p* = 0.007). Visits for slow growth and short stature were instead more frequent in “deferrable” and “planned” referrals compared to “urgent” ones (*p* = 0.04).

The distribution of the reasons for referral, according to priority ranks, is shown in Figure 2. The majority of “urgent” and “deferrable” visits were due to suspected precocious puberty, slow growth/short stature, thyroiditis/hypothyroidism, and precocious pubarche/adrenarche. Those for suspected precocious puberty were more frequent among “urgent” referrals compared to “deferrable” and “planned” ones (*p* = 0.007). Visits for slow growth and short stature were instead more frequent in “deferrable” and “planned” referrals compared to “urgent” ones (*p* = 0.04).

#### Sex Differences

**Figure 3:**
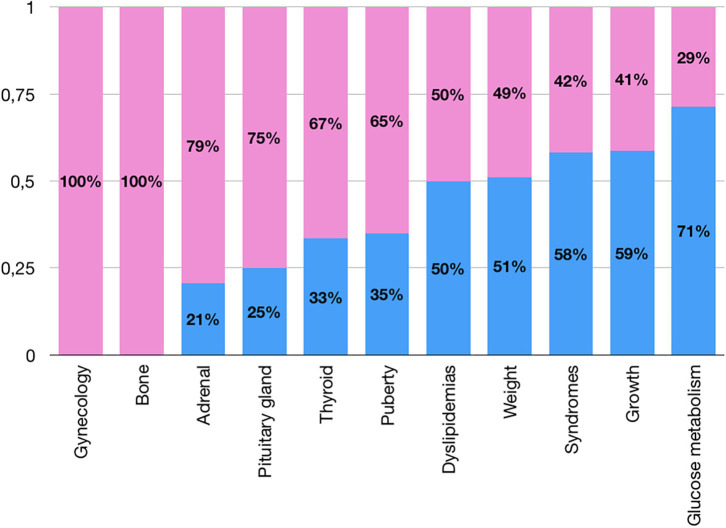
Sex differences in prevalences for cumulative groups of referrals (pink: females; blue: males).

The distribution between sexes in all cumulative groups of referrals is shown in Table 1 and Figure 3.

##### Adrenal

**Figure 4:**
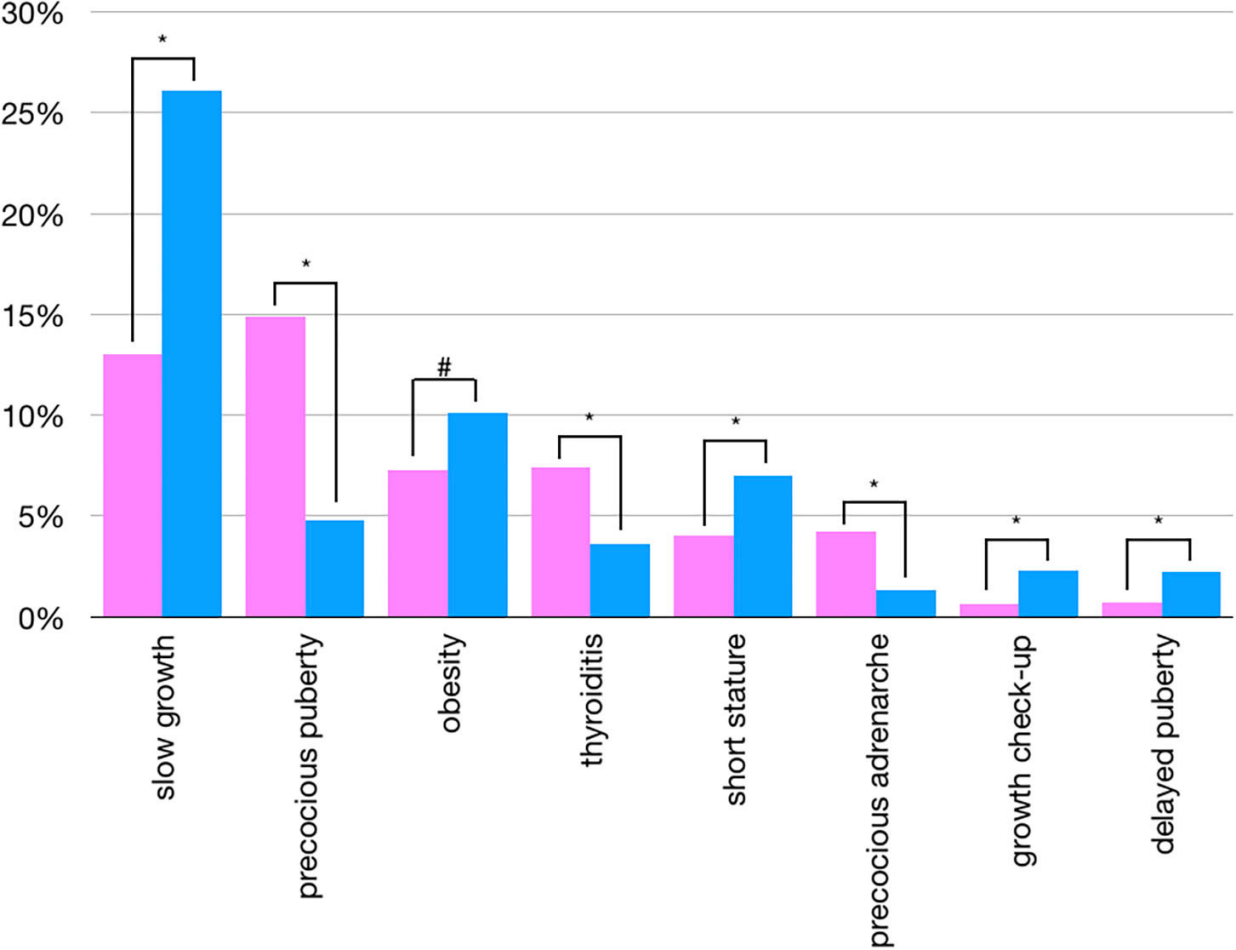
Sex differences in overall prevalences of single referrals (pink: females; blu: males) - only statistically significant data are reported (*p < 0.01 - ^#^*p* < 0.05).

Most of the patients referred for “Adrenal” issues were females (79%), principally because of precocious adrenarche, with an overall prevalence of referrals for this issue in females significantly higher than in males (4.2 vs. 1.3%, *p* < 0.01; Figure 4).

##### Thyroid

A predominance of female patients was found in every single referral reason for thyroid issues, apart from congenital hypothyroidism, where the rate was 1:1 between male and female. The overall prevalence of referrals for thyroiditis in females was significantly higher than in males (7.4 vs. 3.6%, *p* < 0.01, Figure 4).

##### Puberty

More than half of the female “Puberty” referrals were due to suspected precocious puberty (67%), compared to 27% in males, and the overall prevalence of referrals for precocious puberty in females was significantly higher than in males (14.9 vs. 4.8%, *p* < 0.01; Figure 4). While 81% of precocious puberty referrals were females, 69% of referrals for delayed puberty were males, with an overall prevalence of referrals for delayed puberty in males significantly higher than in females (2.2 vs. 0.7%, *p* < 0.01; Figure 4).

##### Weight

Patients referred for obesity and overweight were equally distributed between males and females, although the overall prevalence of referrals for obesity in males was significantly higher than in females (10.1 vs. 7.3%, *p* < 0.05; Figure 4).

##### Syndromes

Among patients referred for a syndrome with presumed endocrinological problems (i.e., CHARGE, Down, Kallmann, Noonan, Prader-Willi, Turner), 42% were Klinefelter syndromes (8.4 years, IQR 5.3–10.6), explaining the predominance of males.

##### Growth

The overall prevalence of referrals for slow growth and growth check-ups were significantly higher in males compared to females (26.1 vs. 13% and 2.3 vs. 0.6%, respectively, *p* < 0.01, Figure 4).

#### Age Differences

Precocious puberty and precocious adrenarche in females were referred at an earlier age compared to males, as expected (Table 3). However, 41% of female and 25% of male referrals for precocious pubarche/adrenarche were sent after the age of 8 and 9 years, respectively, and 56% of females and 61% of males were referred for precocious puberty after the age of 8 and 9 years, respectively.

**Table 3:** Statistically significant differences in age at referral between sexes.

**Referral reasons**	**Females**	**Males**	***P***
	**Median (IQR)**	**Median (IQR)**	
Precocious puberty	8.2 (7.5–9.0)	9.4 (8.5–10.2)	<0.01
Slow growth	10.9 (6.4–12.4)	11.5 (8.3–13.5)	0.01
Hypothyroidism	13.7 (9.1–16.5)	11.2 (5.9–14.0)	0.01
Precocious adrenarche	7.5 (6.8–8.5)	8.7 (8.2–9.1)	0.02

Among delayed puberty referrals, 35% of males were younger than 14 years of age, and 22% of females younger than 13.

Males were referred for hypothyroidism at an earlier age if compared to females, but later than them for slow growth (Table 3).

Females were referred earlier than males for obesity (F 10.2 years, IQR 7.6–14.0; M 11.7, IQR 11.7, 9.6–13.2), although this difference was not significant (*p* = 0.07).

In obesity/overweight, 22% of patients were referred before the age of 8.5 years, while 35% after the age of 12.5 years.

#### Prevalence of Pathological Findings Among Referred Cases, Agreement Between Referral and Final Diagnosis, Treatment and Follow-Up

Overall, in 70% of the visits (*n* = 1,524), diagnostic tests were prescribed. In 814 cases (38% in relation to all the visits, 53% about the prescribed tests) there was a pathological finding; 55 cases were inconclusive for failure to perform planned examinations due to parents' refusal. While in 2013, 53% of patients sent with “urgent” referral had pathological findings, in 2018, this rate dropped to 26%.

**Figure 5:**
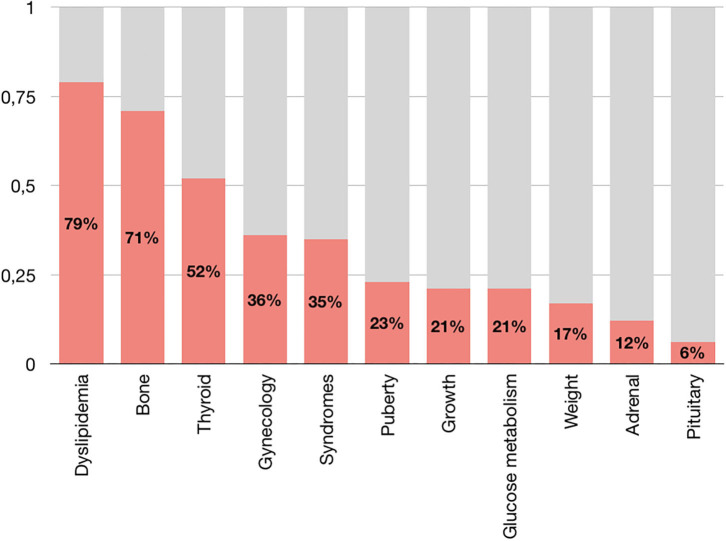
Prevalence of pathological findings in cumulative groups of referral.

The cumulative groups in which there was a majority of visits with a pathological finding were “Dyslipidemia” (79%), “Bone” (71%), and “Thyroid” (52%) (Figure 5).

For 86 patients (4%) the final diagnosis did not agree with the reason for referral (e.g., in 61 the final diagnosis was obesity, starting from a referral of gynecomastia, hypothyroidism, micropenis).

Treatment (pharmacological or behavioral) was prescribed in 35% of cases, and 67% returned at least for one follow-up visit.

##### Thyroid

Patients referred for “thyroid” problems had pathological findings in 52% of the cases, received treatment in 31%, and were followed-up in 60%. The conditions with a higher rate of pathological findings were thyroiditis (76%) and congenital hypothyroidism (90%). In case of nodules, almost all patients performed additional tests (i.e., FNAB), and in a quarter of the cases, the findings were pathologic (≥Tir3 in the classification for thyroid cytopathology). No pathologies were found in referrals for high fT3 or cysts.

##### Puberty

The overall rate of pathological findings in the “puberty” group was 23%. While in males, 43% of patients referred for precocious puberty had pathological findings, in females it was 29%. Patients referred for precocious thelarche had true precocious puberty only in 11% of the cases, and 82% did not even require additional tests. The 8% of patients referred for gynecomastia showed pathological results which—however—were related to complicated obesity rather than to gynecomastia itself.

##### Growth

In the “Growth” group, 71% of the patients had some tests performed, 21% had a final report of pathological findings (29% of the tested patients), and 19% received treatment; 73% were followed-up.

##### Weight

Among patients referred for weight problems, 67% performed additional tests, and 17% had a pathological finding (e.g., impaired glucose tolerance, dyslipidemia), with no differences between males and females; 88% received treatment (pharmacological or behavioral), and 60% attended the follow-up visits. None of the patients referred for underweight had pathological findings.

##### Adrenal

While 65% of patients referred for “adrenal” problems performed tests, only 12% overall had pathological findings, 17% received treatment, and 57% came afterward for a follow-up. The rate of pathologic findings was high in patients referred for hirsutism (42%) and hypertrichosis (50%) and low in those referred for precocious adrenarche (5%) or pubarche (6%), where the real pathology (e.g., precocious puberty or morbid obesity) were often not the reason for the referral. None of those referred for adult body odor and hypercortisolism (*n* = 15) had pathological findings.

## Discussion

In this retrospective study, we comprehensively analyzed 1930 first visits in the pediatric endocrinology outpatient clinic of a tertiary center over 6-years, with a focus on the reasons for the referral and their features. To our knowledge, this is the first study that comprehensively considered all the fields of pediatric endocrinology at the same time over such an extended period of time.

Overall, the vast majority of first visits (83%) were related to four main groups (growth, puberty, thyroid, and weight). The most frequent reasons for referral were slow growth (19%), precocious puberty (11%), and obesity (8%).

Despite the demographic decline in our country (10), which resulted in fewer children over time, there has been an upward trend in visits, which has almost doubled their number during the study period. The increase is not a problem in itself and could be referred to an increased attention in endocrine issues by FP.

Regarding the assignment of priority ranks, the majority of “urgent” or “deferrable” visits were due to reasons that might require quick assessment (precocious puberty/adrenarche/pubarche, slow growth, thyroid problems). On the contrary, a substantial part of the visits did not need a compelling evaluation by specialists (e.g., 16% of “deferrable” and 8% of “urgent” visits were due to overweight/obesity and 4% of both “urgent” and “deferrable” visits were due to subclinical hypothyroidism). These data underline the need to fix rules for priority ranks between FPs and specialists in order to avoid inappropriate “urgent” referrals.

Sex differences were found in some referrals, as expected: a female prevalence was found in referrals for precocious puberty (11), thyroiditis (12), precocious adrenarche (13), while the male one was found in delayed puberty (14) and in the “Growth” group (15, 16). We know that these established sex differences in referrals may not represent a true difference in the incidence of the diseases, but rather due to independent issues (e.g., the difficulty of detecting the onset of puberty in males, overeager evaluations of healthy boys who do not appear to be tall enough) (17).

For the age of referral, in precocious puberty visits, more than half of patients were referred after the age that defines the precocity. For referrals of precocious pubarche/adrenarche, two out of five females and one out of four males were sent, respectively, after 8 and 9 years. Similarly, 22% of females and 35% of males were sent for delayed puberty, before the age that defines delay.

The median age at referral, for growth problems, was rather old (11 years). Although the late diagnosis is common in growth disorders (18, 19), it should be made as early as possible, since efficacy of treatment, when needed, is increased if started at a younger age (20). The majority of cases (three out of four) of overweight/obesity referrals were late (11 years), which is not particularly positive nor effective because children came to visit too late compared the onset and when comorbidities were already present. A previous US study showed that 80% of the children had become obese before the age of 6 years and were referred more than 4 years later (5), which is consistent with our findings. There are recommendations against a routine laboratory evaluation for endocrine causes of obesity in prepubertal to mid-pubertal children unless the child's linear growth is attenuated (21). Therefore, FP should use a life-long longitudinal approach to early help identify children at the risk of obesity, to address the prevention efforts on the family dynamics, dietary, and activity behaviors (22).

Overall, the prevalence of pathological cases among those reported was 38%. While in 2013, more than half of “urgent” visits presented pathological findings, in 2018, only one in four patients sent to the specialist was pathological. Moreover, a small part of referrals (4%) had pathological results that did not agree with the reason for sending.

In the “Growth” group there was a high frequency of referrals, in sharp contrast with the low presence of actually pathological cases (one out of five), which is consistent with other reports (23); moreover, almost three out of 10 children did not require any test.

The “Puberty” group is less than one-fourth of the actual pathological cases (24). Among precocious thelarche referrals, only 11% had true precocious puberty, and a diagnosis of physiologic event can be made in more than 80% of the cases without any further tests. Also, gynecomastia referrals were mostly due to physiological peripubertal events and often misdiagnosed (i.e., adipomastia in obesity) (25). Although a small number of children had a disorder requiring thorough testing and treatment, the vast majority of patients had benign, normal variants of puberty, some of which can be followed by FP without immediate testing or referral.

Overall, many relatively healthy children or children with problems that do not have clinical-pathological significance (e.g., premature isolated thelarche/pubarche, gynecomastia), that can be initially managed in a primary setting (i.e., overweight/obesity, subclinical hypothyroidism) were sent for specialist examination. For example, premature isolated thelarche or subclinical hypothyroidism could be easily monitored by FP, without the need for specialist consultation, unless relevant symptoms and clinical signs appear, therefore requiring expert consultation and appropriate treatment (26, 27). However, sometimes the difference between subclinical or para-physiological conditions and pathological ones may not be clear to a FP, therefore referral may be justified. Pediatric endocrinologists' reassurance may also be needed by a FP to persuade the family about the benignity of a condition.

A potential limitation of this study is based on data collected from a single-center, therefore, patterns and features of referrals may be related to the local system. However, the main findings on prevalence, female/male ratio, and age at referral reported in our study are consistent with other studies in the literature, suggesting that this cohort is not very different from those reported in previous single reports. Another possible limitation is the possible lack of generalizability in countries other than Italy, particularly to health systems without primary care gate keepers.

On the other hand, to our knowledge, this is the first study that has simultaneously analyzed the features of an entire pediatric endocrinology over more than 6 years: it does not just represent a starting point for guiding public health interventions, coordinating specialist care, directing targeted education to FPs, and avoiding inappropriate referrals, but it provided data on the spectrum of all referrals to better organize the outpatient clinics and the education of students/residents in every context.

## Conclusion

The present study showed that the number of pediatric endocrinology outpatient visits has almost doubled over 6 years. The economic impact should not be underestimated: in a public health care system, like the Italian one, a careful cost management is crucial to ensure its sustainability. Inappropriate referrals lead to unnecessary expenses, including journeys and fees for the families and delays in diagnostic-therapeutic processes for those patients who really need pediatric endocrinologist's consultation. A balance is necessary to ensure that patients who need specialist visits, receive them in need get referred, and healthcare systems are not overburdened with unnecessary referrals.

To improve the quality of referrals to our pediatric endocrinology outpatient clinic, we will target continuing medical education of primary care:

1. to better define which are the referral reasons for which an “urgent” or “deferrable” priority rank is appropriate and those who are not;
2. to ask FP a more extended observation period for subclinical or para-physiological conditions;
3. to set the appropriate timing for referring, according to the definitions of pathologies or the best time to intervene.

## Data Availability Statement

The raw data supporting the conclusions of this article will be made available by the authors, without undue reservation.

## Ethics Statement

Ethical review and approval was not required for the study on human participants in accordance with the local legislation and institutional requirements. Written informed consent to participate in this study was provided by the participants' legal guardian/next of kin.

## Author Contributions

EBe conceptualized and designed the study, drafted the initial manuscript, designed the data collection instruments, collected data, and reviewed and revised the manuscript. LM carried out the statistical analysis and reviewed and revised the manuscript. MP, BB, GTa, and MC collected data and reviewed and revised the manuscript. EF and EBa conceptualized and designed the study and critically reviewed the manuscript for important intellectual content. GTo conceptualized and designed the study, drafted the initial manuscript, coordinated and supervised data collection, and reviewed and revised the manuscript. All authors approved the final manuscript as submitted and agree to be accountable for all aspects of the work.

## Conflict of Interest

The authors declare that the research was conducted in the absence of any commercial or financial relationships that could be construed as a potential conflict of interest.
